# Experimental Models for Studying HPV-Positive and HPV-Negative Penile Cancer: New Tools for An Old Disease

**DOI:** 10.3390/cancers13030460

**Published:** 2021-01-26

**Authors:** Beatriz Medeiros-Fonseca, Antonio Cubilla, Haissa Brito, Tânia Martins, Rui Medeiros, Paula Oliveira, Rui M. Gil da Costa

**Affiliations:** 1 Centre for the Research and Technology of Agro-Environmental and Biological Sciences (CITAB), University of Trás-os-Montes and Alto Douro, UTAD, 5001-801 Vila Real, Portugal; fonsecabeatriz@live.com.pt (B.M.-F.); taniam@utad.pt (T.M.); pamo@utad.pt (P.O.); 2 Instituto de Patología e Investigación and Universidad Nacional de Asunción, Asunción, Paraguay; antoniocubillaramos@gmail.com; 3 Maranhão Tumour and DNA Biobank (BTMA), Post-graduate Programme in Adult Health (PPGSAD), Federal University of Maranhão (UFMA), São Luís 65080-805, Brazil; haissa.brito@ufma.br; 4 Veterinary Sciences Department, University of Trás-os-Montes and Alto Douro, UTAD, 5000-801 Vila Real, Portugal; 5 Molecular Oncology and Viral Pathology Group, CI-IPOP, IPO-Porto, 4200-072 Porto, Portugal; ruimedei@ipoporto.min-saude.pt; 6 Faculty of Medicine, University of Porto, 4200-319 Porto, Portugal; 7 Virology Service, IPO-Porto, 4200-072 Porto, Portugal; 8 Biomedicine Research Center (CEBIMED), Faculty of Health Sciences, Fernando Pessoa University, 4249-004 Porto, Portugal; 9 Laboratory for Process Engineering, Environment, Biotechnology and Energy (LEPABE), Faculty of Engineering, University of Porto, 4200-465 Porto, Portugal

**Keywords:** penile cancer, HPV, animal model, mouse model, cell line

## Abstract

**Simple Summary:**

Penile cancer is an uncommon and understudied malignancy that is most commonly diagnosed in developing countries. Therapeutic advances have been slow, in part due to the lack of in vitro and in vivo models for testing new drugs before performing clinical trials. Recently, this difficulty has been partly overcome and multiple new pre-clinical models were reported. These important developments will help develop new therapies for penile cancer patients. The present review summarizes and discusses the available data concerning the pre-clinical models of penile cancer and their uses. Comparisons are drawn between different models, allowing researchers to choose the most adequate setting for their experiments. The remaining gaps in this array of penile cancer models are also discussed, in particular the lack of models for studying metastatic disease and cell lines representing tumors associated with human papillomavirus.

**Abstract:**

Penile cancer is an uncommon malignancy that occurs most frequently in developing countries. Two pathways for penile carcinogenesis are currently recognized: one driven by human papillomavirus (HPV) infection and another HPV-independent route, associated with chronic inflammation. Progress on the clinical management of this disease has been slow, partly due to the lack of preclinical models for translational research. However, exciting recent developments are changing this landscape, with new in vitro and in vivo models becoming available. These include mouse models for HPV^+^ and HPV^−^ penile cancer and multiple cell lines representing HPV^−^ lesions. The present review addresses these new advances, summarizing available models, comparing their characteristics and potential uses and discussing areas that require further improvement. Recent breakthroughs achieved using these models are also discussed, particularly those developments pertaining to HPV-driven cancer. Two key aspects that still require improvement are the establishment of cell lines that can represent HPV^+^ penile carcinomas and the development of mouse models to study metastatic disease. Overall, the growing array of in vitro and in vivo models for penile cancer provides new and useful tools for researchers in the field and is expected to accelerate pre-clinical research on this disease.

## 1. Introduction

Penile cancer is a rare disease in Europe and in North America, where it accounts for only 0.4% to 0.6% of all cancers [1]. The incidence is higher in less-developed countries, where it can reach up to 6.15 new cases per 100,000, according to recent data [2]. In total, 36,068 new cases were estimated to have occurred worldwide in 2020 [3]. Penile cancer predominantly occurs in men between 50 and 70 years of age, although younger individuals may also be affected. This disease has been associated with risk factors such as human papillomavirus (HPV) infection, phimosis, low socioeconomic status, poor hygiene and smoking [1]. Infection by HPV is a major risk factor and two pathways leading to penile carcinogenesis are currently recognized: one is associated with HPV infection, while the other is HPV-independent and has been linked with chronic inflammatory conditions such as chronic balanoposthitis caused by phimosis [4,5]. Phimosis is the difficulty in exposing the penile glans due a stenotic foreskin, which impairs personal hygiene of the penis. It should be noted that the presence of an intact foreskin by itself is a risk factor for penile cancer and that circumcision reduces this risk [6,7]. Neonatal circumcision is associated with lower rates of penile cancer (e.g., in Jewish communities) [7,8,9,10], which is ascribed to a reduction in smegma accumulation [6,7,11,12]. Accumulation of smegma— composed of exfoliated epithelial cells, oils and fats—leads to genital inflammation and can be aggravated by poor hygiene [6,7,11,13]. Smoking is another important risk factor, and smokers are three to four times more likely to develop penile cancer compared with non-smokers [7,9]. Mutagenic chemicals from tobacco are found in smegma and are believed to promote DNA damage and cell transformation of the penile epithelium [7,12,14]. Penile carcinogenesis begins with small lesions—classified histologically as penile intraepithelial neoplasia (PeIN)—on the penile glans or foreskin. If left untreated, PeIN can evolve into invasive squamous cell carcinomas that may require extreme surgical approaches [15]. Most human penile carcinomas are squamous cell carcinomas, and among these, a number of special subtypes are recognized [16]. The bimodal carcinogenic pathway, whether HPV-dependent and non-HPV-dependent, was recognized in previous seminal studies from our group [17,18]. Non-HPV-related penile carcinomas comprise approximately one half of all human penile carcinomas [19]. Morphologically these are keratinizing low grade neoplasms except for sarcomatoid carcinomas, which are non-keratinized and show a high histological grade. The usual or conventional squamous cell carcinoma is the most common subtype. There are other clinically and morphologically distinctive variants, such as the pseudohyperplastic carcinoma [20] occurring in older patients with longstanding lichen sclerosus and the pseudoglandular carcinoma [21], an aggressive variant which simulates adenocarcinomas. Verrucous carcinoma is a non-metastasizing low grade neoplasm in its pure version, but when mixed with the usual squamous cell carcinoma is classified as hybrid verrucous carcinoma and has metastatic potential [16,22]. Carcinoma cuniculatum is a rare, low grade, deeply invasive tumor, with a labyrinthine growth pattern and no metastatic potential which is considered a variant of verrucous carcinoma [23]. Other tumor types usually not related to HPV are the papillary not otherwise specified (NOS) [24], of good prognosis, the adenosquamous carcinoma [25] and the sarcomatoid carcinomas [26], the latter of which has the worst prognosis among all penile carcinomas. There are mixed carcinomas where more than one type is present in the same specimen [16]. HPV-related penile squamous cell carcinoma has distinctive morphological features which can be most often recognized with routine pathological stains. These comprise between 30% and 50% of all penile carcinomas and occur in patients about 10 years younger than those with non HPV related neoplasms [15]. The most common penile carcinomas are the basaloid [27] and the condylomatous (warty) carcinomas [28]. The former is non-verruciform and endophytic, with a high rate of nodal metastasis while the latter is a verruciform exophytic tumor, which is rarely associated with regional spread. There are other variants like the warty-basaloid carcinoma [29], usually displaying equal parts of basaloid and warty carcinoma, and the less common papillary-basaloid [30] and clear cell carcinomas [31]. Very unusual HPV-related tumor variants are the lymphoepithelioma-like [32] and the medullary carcinoma [33]. Penile sarcomas are vanishingly rare and usually affect corpora cavernosa. Leiomyosarcoma is the most common type [34]. Metastatic tumors affecting the penis are rare, with only 529 cases reported up to 2016. Prostate and bladder carcinomas are the most common primary sites (71%), followed by the gastrointestinal tract (19%), lungs (5%) and bone (1%). Corpora cavernosa is the most common site affected by metastatic carcinomas on the penis, but other areas like the corpus spongiosum, penile fascia or foreskin may also be involved [16].

Penile tumors will grow slowly along the surface of the penile mucosa and skin, covering first the glans and/or the foreskin before invading the erectile corpora spongiosa and cavernosa as well as the penile shaft. Ulcerative lesions have been found to metastasize more readily than exophytic lesions to the regional lymph nodes [7]. Buck’s fascia covers the corpora cavernosa and spongy body, and the likelihood of metastasis increases once this structure is compromised [7]. Importantly, there is a predictable and gradual pattern for the metastatic progression of penile cancer [35]. Lymphatic drainage occurs primarily for superficial inguinal lymph nodes, then to deep inguinal lymph nodes and to the external iliac lymph nodes in the pelvis, and metastases develop in this order [9,35,36,37]. Afterwards, other (e.g., para-aortic, mediastinal) lymph nodes are affected and the regional dissemination to the skin of the pubis and groin, as well as the direct invasion of the prostate, scrotum and testis, may occur. In later stages, metastases to multiple sites occur, preferentially to the liver, lungs and heart, and finally in multiple sites [35]. Lymphadenectomy, radiotherapy and perioperative chemotherapy are therapeutic options for patients with regional lymph nodes metastases [10]. The surgical treatment for many patients with advanced penile cancer is still penectomy and urethrostomy, but these techniques have devastating psychological effects [38,39]. Organ-sparing techniques have been developed that allow the treatment and preservation of the penis in specific cases [38,39]. A number of models for penile reconstruction have also been reported using bioengineering approaches [40,41,42,43,44,45,46,47], and are promising alternatives for restoring penile anatomy and physiology following surgery. The prognosis for advanced disease is dismal and platinum-based chemotherapy for patients with metastatic penile cancer only affords 6 to 12 months of median overall survival [48], thereby necessitating urgent improvements. As was recently reviewed [49], a number of ongoing clinical trials are striving to test the safety and efficacy of new therapies for penile cancer, including drugs belonging to innovative drug classes such as immune checkpoint inhibitors. This renewed interest in penile cancer therapy and the recent development of the first mouse models for penile cancer [50,51] brings hope to researchers in this field, as these new tools should help with accelerating translational research and achieving more effective therapies for penile cancer patients. New cells lines are also available for in vitro drug screens [52]. This review brings together those recent developments, discussing in vivo and in vitro models of penile cancer and their potential applications.

## 2. Role of HPV in Penile Cancer

The incidence of penile cancer is higher in less developed countries (2–4 cases per 100.000 habitants) than in developed countries (0.5–1 per 100.000 habitants) and this malignancy can account for up to 10% of male cancers in some parts of Africa, South America and Asia [53,54]. PeIN is the most common clinical presentation in countries with low incidence of penile cancer; the opposite occurs in countries with a high incidence of penile cancer, where invasive penile carcinoma is the typical presentation. The most likely cause for this geographic variation is the earlier diagnosis and treatment of precancerous lesions in developed countries compared with patients, and clinical neglect and late diagnosis in less developed countries. About half of invasive penile carcinomas are HPV related. HPV is transmitted by direct contact and infects keratinocytes in the basal layer of the stratified squamous epithelia of the skin—the epidermis—and mucous membranes, such as the penile mucosa, as well as other well-characterized ano-genital sites like the uterine cervix and the oral and oropharyngeal mucosa [55]. High-risk HPVs, such as HPV16 and HPV18, which are associated with cervical and other ano-genital cancers, and a growing subset of oropharyngeal cancers, present the E6 and E7 oncogenes, which exert critical cell transforming functions, as was recently reviewed [56]. Briefly, the E6 and E7 genes encode the E6 and E7 oncoproteins, which target two key tumor suppressor proteins: p53 and the retinoblastoma protein (pRb). The E6 and E7 oncoproteins of high-risk HPVs induce the degradation of their respective targets, leading to uncontrolled cell proliferation, loss of cell differentiation and resistance to apoptotic stimuli [7,8,56]. The E5 oncogene of high-risk HPVs also contributes to cell transformation, but its role in HPV-driven carcinogenesis is less well understood [56]. While the activity of these viral oncoproteins promotes the development of the major hallmarks of cancer, other factors are also involved in HPV-induced carcinogenesis, including changes in cellular epigenetic regulation [57] and in the local tissue microenvironment [58], as well as environmental exposure to chemical co-carcinogens [59]. Whereas the causes of penile cancer are not entirely understood, two causal pathognetic pathways have been suggested that are either HPV related or non HPV related [17,18]. This view resulted in the 2016 WHO pathological classification of penile carcinomas in HPV dependent and non HPV dependent [60], based on their histological features (e.g., the presence of koilocytes) and molecular features (e.g., the presence of HPV DNA and overexpression of p16^INK4A^) [15]. HPV 16 is by far the most common genotype in penile cancer [19]. Two large studies reported estimates concerning the proportion of HPV-related and HPV-independent penile cancers [4,5]. Alemany et al. (2016) conducted an international study using over 1000 samples from 25 countries and identified HPV DNA in 33% of cases [4]. Olesen et al. (2018) performed a meta-analysis of studies reporting prevalence of HPV DNA and the surrogate marker p16^INK4A^, which is overexpressed in cells where pRb has been inactivated by the E7 oncoprotein [5]. These authors estimated that 50% of cases show HPV DNA and 42% were positive for p16^INK4A^. The authors also reported that more than 80% of PeIN cases were positive for HPV. Interestingly, a study comparing the incidence of HPV in PeIN and penile cancer in an endemic area (Paraguay) and a non-endemic area (France), found that HPV-related PeIN was more prevalent in the non-endemic area [61]. Discrepancies in the proportion of HPV-positive cases may be explained by geographic variations and by the use of different methods for studying the presence of HPV DNA and the expression of p16^INK4A^. Estimating the proportion of HPV-related penile cancers is an important priority, as HPV infection is preventable through vaccination, and effective vaccination strategies might prevent this subset of penile cancers [62,63,64]. Additionally, the presence of HPV is associated with specific molecular signatures, including hypermethylation of genes such as CD70, HN1, FZD5, FSCN1 and PRR16 [65] and may have an impact on the biological behavior of penile malignant [66,67] and pre-malignant lesions [68] and on the prognosis of cancer patients [66], although its clinical significance still requires additional clarification [69].

## 3. Cell-Based Models of Penile Cancer

A number of cell lines representing primary penile squamous cell carcinomas and lymph node metastases have been reported and are summarized in Table 1. The first successful efforts to culture penile cancer cells in vitro were reported in the 1960s [70], and since 2010, a growing number of cell lines—some of which extensively characterized at the molecular and morphological levels—has been described. All of these are able to grow as xenografts in mice (see Table 1), allowing researchers to use those cells for in vivo applications. While the pre-2000 studies do not report the HPV status of cells lines or their original tumors, it is remarkable that all cell lines with defined HPV status are HPV-negative. This lack of representation of HPV-positive disease constitutes a major gap in the array of cell-based models available and additional efforts are needed to develop cell lines representing HPV-positive tumors. The use of these cell-based models for basic and translational research increased in recent years, with multiple publications reporting the use of cell lines developed by Chinese researchers at the Sun Yat-Sen University Cancer Center. Zhou et al. (2018), using a panel of five cell lines, reported their sensitivity to cisplatin and resistance to therapy directed against the epithelial growth factor receptor 1 (EGFR), in association with frequent *EGFR* amplification [52]. The Penl1 cell line was also used to study the effects of overexpressing inhibitor of DNA binding 1 (ID1), which was found to promote tumor progression [40]. In the same year and using the same cell line, the overexpression of the carcinoembryonic antigen-related cell adhesion molecule 19 (CEACAM19) was found to promote tumorigenesis via activation of small mothers against decapentaplegic (Smad) 2 and 3 and increased metalloproteinase (MMP) 2 and 9 secretion [41]. Conversely, knockdown of the insulin-like growth factor binding protein 2 (IGFBP2) suppressed cell growth, clonogenesis and migration [42,71]. Knockdown of chemokine C-X-C motif ligand 13 (CXCL13) in the Penl1, Penl2, 149RCa and LM156 cell lines suppressed cell proliferation and survival, clonogenesis, migration and invasion via reduced MMP2/9 secretion [43]. These are recent examples of how such well-characterized cell lines can prove to be useful for researchers wishing to explore the biopathology of penile cancer or to test potential new therapies, either using in vitro settings or xenograft models. It is also worth mentioning that the first mouse syngeneic cell-based models were recently reported [50]. These syngeneic models offer an opportunity to study penile cancer cells in a fully immunocompetent murine host and may complement studies done in vitro or with xenografted human cells. The authors established two cell lines from murine penile carcinomas occurring in C57Bl/6 mice that were either *Smad4*/*Apc* null (SA1 cells) or *Smad4*/*Apc*/*Pten* null (SAP1 cells) (see Section 4.2 where these mouse models are dealt with in detail). The sensitivity of these cells towards cisplatin and a panel of 42 small molecules selected based on previous proteomic analysis was also tested [50]. This growing array of human and mouse cell-based systems offers opportunities to explore basic and translational aspects of penile cancer and will be essential to drive pre-clinical research in coming years.

## 4. In Vivo Animal Models

The first mouse models for penile cancer were recently reported using either mice transgenic for HPV16 [51] or knockout mice for the tumor suppressor genes Smad4 and Adenomatous polyposis coli (Apc), with or without deletion of Phosphatase and tensin homolog (Pten) [50,78]. These models represent major advances for pre-clinical research on penile cancer and will be discussed in detail in the next paragraphs. Table 2 summarizes the main features of the in vivo models for studying penile cancer that were reported so far.

### 4.1. Mouse Penis: Anatomy and Histology

**Figure 1:**
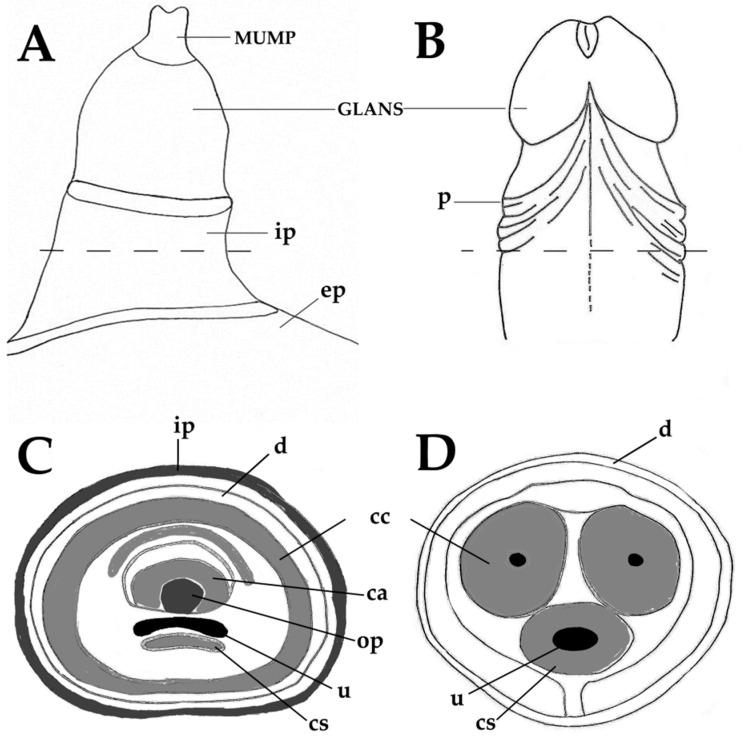
Anatomy of the adult murine (**A**,**C**) and human (**B**,**D**) penis. Male Urogenital Mating Bulge (MUMP)-male urogenital mating bulge, p-prepuce, ep-external prepuce, ip-internal prepuce, ca-MUMP cartilage, cc-corpus cavernosum, cs-corpus spongiosum, d-dartos, op-os penis, u-urethra.

The choice of an animal model is based on similarities with human anatomy, physiology and pathology. When considering mouse models of penile cancer, it is necessary to consider similarities and differences between these animals and humans. Keeping this in mind, the next paragraphs and Figure 1A–D present a short comparison between the human and mouse penis, before discussing the new mouse models of penile cancer. As in men, the penis of mice has the main functions of urination, copulation and placement of sperm in the female reproductive tract. In mice, the primordial tubercle that ultimately originates the male penis and the female clitoris is formed between the 12th and 16th gestational days and the differentiation of the female and male organs occurs from the 16th day onwards [80]. After birth, the morphological distinction of female and male external genitalia becomes feasible at approximately 4 weeks of age [80]. The mouse penis consists of a proximal body and distal glans that connect in a right-angle curve [80]. The penile body begins near the pelvic outlet, where the urethra bends at a right angle. At this point, the corpora cavernosa leaves the penile body and diverge laterally to attach to the pubic bone. Thus, the penile body contains the urethra and the right and left cavernous body that will merge in the midline. The penile body ends distally in a right-angle curve, where the penis body joins the glans. The glans contains the glandular spongiosa and cavernosa bodies, the urethral spongiosa body, and the skeletal elements, including a transverse element containing bone and a growth plate of hyaline cartilage and a distal element composed of fibrocartilage that also ossifies after puberty. These skeletal elements allow for the necessary rigidity for a successful mating and account for a major anatomical difference compared with the human penis [54]. The surface of the penile glans is covered by a stratified squamous epithelium showing light keratinization, which is histologically similar to that of the human penis, where most penile squamous cell carcinomas arise. This is an important similarity, as genetic modifications intended to induce squamous cell carcinoma in mouse models are all directed at this epithelial layer. However, this epithelium contains epithelial spines, which constitute another morphological difference when compared with the human penile mucosa. The distal part of the glans is designated the Male Urogenital Mating Bulge (MUMP) [81]. The central element of MUMP is a 1.7 mm long fibrocartilaginous piece. Centrally, the penile bone extends from the right-angle curve of the penis to the MUMP and measures about 3.8 mm in length. In its distal portion, the bone is overlapped dorsally by the MUMP fibrocartilage (Figure 1A,C). The glandular cavernous body is circumferential, while the urethral spongiosa body is linear, runs ventrally to the urethra and appears to be homologous to the corpus spongiosum in humans (Figure 1C–D) [80]. Different to humans, mice have 2 preputial anatomical compartments, the inner covered by smooth non hairy mucosa and the outer covered by hairy skin (Figure 1A–B). The human inner foreskin may be considered an equivalent of the inner mouse foreskin, and the human outer dermal and epidermal foreskin may be equivalent to the mouse outer hairy foreskin. Externalization of the penis in mice can be achieved by applying light abdominal pressure, which allows the glans to leave the external foreskin, extending away from the proximal body. As the glans extends, the inner foreskin protrudes next to it, individualizing the glans, inner foreskin (entirely covered by a keratinizing mucosa) and outer foreskin (covered by haired skin on the external surface) [80]. Human penile anatomy, which is considered to be complex by pathologists, is simpler than that of experimental mice. The glans and inner foreskin surfaces are covered by a stratified squamous epithelium where PeIN develops. Below is the lamina propria, corpus spongiosum or dartos and corpora cavernosa. Tumors progresses vertically along these anatomical levels. In the foreskin, tumors progress from the epithelium to the lamina propria to dartos to dermis to epidermis [54].

### 4.2. HPV-Negative Penile Cancer in SMAD4/APC Double Knockout Mice

Penile squamous cell carcinomas were obtained in C57Bl/6 mice by targeted deletion of the Smad4 and Apc tumor suppressor genes on the penile epithelium. A preliminary report on this model was released in 2017 [78] and a full characterization was recently published, revealing its great potential for pre-clinical research [50]. This model was developed using the androgen receptor (AR)-responsive probasin gene promoter, which is commonly used in mouse models of prostate cancer [82] since the penile epithelium is positive for AR [50]. The probasin gene promoter was used to drive the expression of a Cre recombinase and delete Smad4 and Apc in PB-Cre4+ Smad4^L/L^ and/or Apc^L/L^ mice. The deletion of each gene individually was insufficient to induce cancer, but their co-deletion was found to induce squamous cell carcinoma of the penis at 100% penetrance (median age 17.2 weeks-old). Although the tumors were initially induced via AR-driven mutagenesis, they were found to be AR-independent, and tumor progression was unaffected by castration. Transcriptomic analysis of these lesions confirmed the deregulation of the Wnt/β-catenin and the fibroblast growth factor (FGF) pathways induced by their genetic alterations. Importantly, the penile tumors transcriptome also revealed marked pro-inflammatory signaling pivoted by cyclooxigenase-2 (COX-2) and massive infiltration by immunosuppressive myeloid cells. This animal model was used to test the efficacy of rational drug combinations based on immune checkpoint inhibitors (anti-PD1/anti-CTLA4 antibodies) combined with either the selective COX-2 inhibitor celecoxib or with the multi-target tyrosine kinase inhibitor cabozantinib. The drug combinations proved to be significantly more effective than each drug alone and their anti-tumor effects were associated with reduced infiltration of myeloid cells and regulatory T lymphocytes. These results highlight the potential of this model for developing innovative combination therapies, especially those involving immune checkpoints blockade. Interestingly, celecoxib was also shown to increase the activation of cytotoxic T lymphocytes in a different model of epithelial carcinogenesis induced by HPV16, further supporting the use of COX-2 inhibition for potentiating immunological therapies [83]. Considering the role of Pten in the resistance of penile cancer to platinum-based therapy, the authors also developed a triple knockout, Smad4/Apc/Pten null mouse model. While the double knockout tumors remained sensitive to cisplatin, the triple knockout lesions were largely resistant, corroborating the role of Pten loss in driving resistance to platinum-based therapies in human patients. These findings validate those mouse models for studying strategies to overcome cisplatin resistance. The syngeneic cell lines developed from both the double and the triple knockout models (see the previous Section 3 dealing with cell-based models) are equally powerful tools for translational studies. Importantly, the authors reported that orthotopic injection of these cells lines was able to replicate penile squamous cell carcinoma. This is more realistic than implanting cell-based models heterotopically (most often subcutaneously) and should allow for drug development as well as for identifying key mechanisms involved in the invasion of penile structures. One important caution for researchers working in this field is that these models seem more adequate for representing HPV-negative disease than HPV-positive disease. Although the authors present their mouse models in the context of HPV-positive penile cancer and highlight similarities with HPV-positive disease, several key differences exist: (1) the absence of HPV oncogenes in these mouse models; (2) one key HPV target, pRb, remains present and phosphorylated at high levels; and (3) the gene mutations employed to generate this mouse models are found in HPV-positive and negative tumors and in many other epithelial neoplasms [84,85]. Overall, the models by Huang et al. (2020) are very promising tools to study HPV-negative disease and should be used in combination with other models that can adequately mimic HPV-positive lesions [50].

### 4.3. In Vivo Models for HPV-Positive Penile Cancer

An ideal model for studying HPV-positive penile cancer would be driven by key oncogenes from high-risk HPVs (e.g., HPV16 E6 and E7), would develop lesions specifically at the penis and would reproduce the main morphological and molecular features of HPV-induced carcinogenesis (e.g., basaloid PeIN, HPV-associated SCC subtypes followed by lymph node metastasis) [15]. Parts of these aims have been recently achieved by different research teams using complementary models (Table 2). A recently reported mouse model for HPV-positive penile cancer employs mice carrying the whole HPV16 early region (containing the key oncogenes E5, E6 and E7) in an FVB/N genetic background [51]. In this model, the expression of HPV oncogenes is targeted to basal keratinocytes by the cytokeratin 14 (Krt14) gene promoter [86,87] and the mouse strain is often referred to as K14-HPV16. This mouse strain was originally developed in the 1990s [86,87] and other related strains, carrying only the E6 and or E7 oncogenes, were used to study a number of HPV-induced cancers, including cervical cancer [86] anal cancer [88] oral, esophageal and oropharyngeal cancers [89,90]. These mice are also useful for studying interactions between HPV and the immune system—as they are fully immunocompetent—as well as with hormonal and other microenvironmental factors and with potential chemical co-carcinogens (e.g., tobacco toxins) [83,91,92]. One limitation of this model system is the fact that expression of HPV oncogenes is not regulated by the viral long control region or by cellular mechanisms normally involved in naturally infected patients, instead is driven by the Krt14 gene promoter [86,87]. Another limitation is the widespread occurrence of intraepithelial hyperplastic and dysplastic lesions throughout the skin and keratinizing mucosae, which may reduce the specificity of models intended solely for penile cancer [51]. This difficulty may be partially overcome by obtaining syngeneic cell lines that can be injected orthotopically or heterotopically into matched FVB/N HPV-negative mice. Finally, HPV-transgenic mice are not good models to study the mechanisms of HPV cell entry and infection. Models based on natural infection by the murine papillomavirus (MmuPV1) are a promising alternative in this regard, but no penile lesions caused by mmuPV1 have been reported so far [71,93]. HPV16-transgenic mice were found to develop PeIN and condylomas at 30 weeks-old, but no squamous cell carcinomas [51]. These were obtained at a 29.6% incidence by exposing HPV16-transgenic mice to the tobacco-related carcinogen dimethylbenz(a)anthracene (DMBA) topically in the penile mucosa weekly, for 16 weeks. Importantly, the presence of the HPV oncogenes was associated with deregulated cell proliferation, as assessed immunohistochemically using Ki-67, a marker for proliferative cells. This protein is present in the G1, S, G2 and mitotic phases of the cell cycle, and is used to infer the growth fraction of a given cell population [94,95,96]. The intraepithelial and invasive lesions observed in this animal model closely mimicked the histological features of HPV-positive penile lesions in human patients. PeIN lesions predominantly showed basaloid morphology and koilocytosis, typical of HPV-induced pathology. Invasive squamous cell carcinomas also corresponded to histological subtypes usually associated with HPV positivity like basaloid, warty-basaloid and solid medullary-like tumors [15,31,35,97]. DMBA application in this model was not associated with increased incidence of SCC on cutaneous locations as assessed histologically nor with systemic genotoxicity, according to screens using the micronucleus and the comet assays. Importantly, these observations provided the first experimental demonstration of the etiological role of HPV16 in penile cancer. Additionally, this study also supports the role of tobacco toxins as important promoters of penile carcinogenesis. While this model reproduced the whole spectrum of HPV-induced penile lesions, no metastases were observed, limiting its usefulness to study the more advanced stages of the disease. Another interesting model for studying penile cancer induced by papillomaviruses may be found in horses. These animals develop penile papillomas, intraepithelial neoplastic lesions and squamous cell carcinomas which are associated with infection by equine papillomavirus type 2 (EcPV2) [79,98,99,100]. Importantly, some equine penile squamous cell carcinomas are able to metastasize to regional lymph nodes, something that has not yet been observed when studying the newly available mouse models [79]. Interestingly, the characterization of these tumors yielded some markers that are similar to those observed in human patients [49,101]. Suárez–Bonnet et al. (2018) found that COX-2 was neo-expressed in 86% of cases, and the expression was higher in squamous cell carcinomas than in papillomas [49]. In squamous cell carcinomas, E-cadherin was present in 65% of cases, and vimentin was neo-expressed in 65% of poorly differentiated cases [49]. The cytoplasmic expression of 14-3-3σ protein was observed in 42% of squamous cell carcinomas [49]. Pten expression tended to be decreased or lost in squamous cell carcinomas [49], which correlates with cisplatin resistance in human patients and in another animal model, as previously discussed [50]. MMP1 is also expressed in equine penile lesions [100], which is in line with the expression of MMPs reported in human and murine penile [50]. These lesions also show nuclear p53 accumulation, revealed by immunohistochemical techniques, suggesting that p53 gene mutations are common despite a papillomaviral etiology [79]. This may be a specificity of the equine model and deserves further elucidation. Taken together, these observations suggest that equine penile lesions may be a useful spontaneous model of penile neoplasia, from which there is much to be learned.

## 5. Conclusions

The recent development of the first models for studying penile cancer in vivo, along with new cell lines, is paving the way for translational research. These are powerful tools for preclinical studies and should help advancing the development of new therapies for cancer patients. The major remaining gaps are the need for cell lines that represent HPV-positive penile cancer and a greater refinement of HPV-positive in vivo models, including their genomic and transcriptomic characterization and the syngeneic cell-based models for drug tests. In the clinical setting, there is a significant number of ongoing trials aiming to determine the efficacy and safety of new therapies for penile cancer, as recently reviewed [101]. The joint efforts of researchers working on the pre-clinical and the clinical levels will open new horizons of hope for patients suffering from this deadly disease.

## Figures and Tables

**Table 1:** Penile cancer cell lines and their characteristics.

Cell Lines	Tissue of Origin	HPV Status	Morphology	Other Characteristics	References Publication Year
First reported penile cancer cell line	Primary tumor	Not reported	Epithelial	Cytogenetic characterization reported	[70] 1966
TSUS-1	Negative	Not reported	Epithelial	Epithelial morphology, cytogenetic characterization reported, mean doubling time 38 hours	[72] 1983
PCA-5	Negative	Not reported, human herpesvirus detected	Epithelial	Epithelial morphology, cytogenetic characterization	[73] 1987
KU-8	Lymph node metastasis	Not reported	Epithelial	Epithelial morphology, cytogenetic characterization reported, mean doubling time 20 hours, EGFR-positive	[74] 1989
Ki-PeCa-L1, Ki-PeCa-P1	Primary tumor (Ki-PeCa-P1), lymph node metastasis (Ki-PeCa-L1)	Not reported, positive for p16INK4A	Epithelial	Chemokine profiles available	[75] 2012
P5	Negative	Negative	Epithelial morphology but sarcomatoid when cultured in vivo	Genomic and transcriptomic characterization	[76] 2016
Penl1, Penl2, 149RM, 149RCa, LM156	lymph node metastases (Penl1, Penl2, LM156), locally recurrent lesion (149RM) scrotal invasion lesion (149RCa)	Negative	Epithelial	penl2 doubling time: 28 hours, 149RM 26 hours, 149RM and 149RCa 26 hours, LM156 34 hours. All cell lines: genomic characterization available, sensitive to cisplatin, resistant to anti-EGFR therapy	[52,77] 2016, 2018
SA1	C57Bl/6 mouse primary tumor	Negative	Epithelial	Smad4 and Apc null, cisplatin-sensitive. Genomic, methylation and transcriptomic characterization	[50] 2020
SAP1	C57Bl/6 mouse primary tumor	Negative	Epithelial	Smad4, Apc and Pten null, cisplatin-resistant. Genomic, methylation and transcriptomic characterization	[50] 2020

**Table 2:** In vivo models for HPV-positive and HPV-negative penile cancer.

Species/Strain	HPV Status	Genetic Modifications	Other Characteristics	Reference Publication Year
Horse	HPV status: negative but most are EcPV2-positive.	None.	Spontaneous model. Occurs infrequently in horses. Intraepithelial and pre-malignant lesions: papillomatous lesions. Metastasis: yes, to lymph nodes.	[79] 2014
C57Bl/6 mouse	HPV status: negative.	Based on targeted deletion of *Apc/Smad4* with or without *Pten* deletion.	100% SCC incidence. Pten deletion confers cisplatin resistance. Intraepithelial and pre-malignant lesions: not described. Metastasis: no.	[50] 2020
FVB/N mouse	HPV status: Positive for HPV16.	Based on targeted expression of the entire HPV16 early region.	Requires exposure to DMBA.29.6% SCC incidence. Intraepithelial and pre-malignant lesions: yes, condylomas and penile intraepithelial neoplasia. Metastasis: no.	[51] 2020
